# When “lying flat” goes quiet: active and passive pathways linking “Tang Ping” suppression to displaced voice and hidden disengagement

**DOI:** 10.3389/fsoc.2026.1877139

**Published:** 2026-08-25

**Authors:** Hugh Jiliang Liu

**Affiliations:** 1 International Business Unit, Business and Law Cluster, Hanze University of Applied Sciences, Groningen, Netherlands; 2 Department of Psychology, Faculty of Behavioral and Social Sciences, University of Groningen, Groningen, Netherlands

**Keywords:** China, lying flat, psychological reactance, self-determination theory, suppression, Tang Ping, TP discourse, TP practice

## Abstract

Tang Ping (躺平, TP), or “lying flat,” has emerged among Chinese youth as an embedded response to overwork, involution, blocked mobility, and declining confidence that sustained effort will secure adult roles and material stability. This Perspective asks what may happen when institutions and platforms restrict the visibility, legitimacy, or circulation of TP expression without changing the pressures that made it meaningful. It introduces the Tang Ping Differentiated Pathways Framework (TP-DPF), a conceptual model of function-contingent responses to suppression. The framework distinguishes three manifestations of TP: discourse, self-identification, and practice. Its originality lies in combining this manifestation-function distinction with function-contingent divergence after suppression. When TP expression serves active boundary-setting and retains agency, suppression may evoke autonomy-threat appraisal, freedom-restoration motivation, and displaced voice through coded, private, anonymous, or symbolic expression. When TP expression reflects passive, despair-driven withdrawal and low efficacy, suppression may instead reinforce voice-futility appraisal, futility-based withholding, and intensified hidden disengagement. Self-determination theory differentiates the two functions; psychological reactance theory explains the active pathway; and voice-and-silence theory explains the passive pathway. Chinese relational obligations, moralized striving, class-linked resources, and state-platform visibility governance may condition discourse-practice correspondence and post-suppression pathways. The article specifies construct definitions, multilevel boundary conditions, operational propositions, sampling and measurement strategies, instruments, and analytical designs. It concludes cautiously that reduced public TP discourse by itself cannot demonstrate motivational recovery because expression may diverge from practice, active voice may be displaced, and passive withdrawal may deepen.

## 1 Introduction

Tang Ping (躺平, TP), meaning “lying flat,” refers to a countercultural phenomenon among some Chinese young people who communicate, identify with, or enact a reduced commitment to socially prescribed forms of intensive striving. TP is characterized by reducing material desire, limiting participation in high-pressure competition, and refusing the moral imperative to constantly strive (Hsu, 2022). In China, the expression became prominent in relation to “996” work culture, educational and occupational competition, housing pressure, platform-economy insecurity, and “neijuan” or involution. Involution describes escalating competition in which additional effort yields diminishing psychological, social, or economic returns. TP is therefore not merely a private lifestyle preference. It is a socially meaningful response to a perceived motivational trap and to a changing effort-reward contract.

TP may appear in at least three analytically distinct manifestations: TP discourse, referring to what people communicate or symbolize; TP self-identification, referring to whether they explicitly adopt the TP label; and TP practice, referring to what they actually do in work, education, consumption, or family life. Throughout this article, TP expression is used as the umbrella term for TP discourse and communicative self-identification, whereas TP practice refers specifically to enacted behavior. These manifestations may coincide, but they need not. A young person may publicly endorse TP while remaining unable to reduce intensive work, competition, or family obligations; conversely, another may enact withdrawal without publicly adopting the label. Tong and Liu (2024) illustrate this divergence through class-differentiated modes of TP, showing that access to economic, cultural, and social resources may shape whether and how a desire to lie flat can be translated into practice. Accordingly, neither public TP expression nor observable withdrawal should be treated as a direct proxy for TP function.

The most consequential distinction concerns not simply the extent to which an individual appears to “lie flat,” but the psychological function served by a TP expression or practice.Recent research suggests that TP may involve both active and passive functions, although this distinction remains theoretically useful rather than empirically settled (Sun, 2025; Zhang et al., 2025). Active TP refers to a self-endorsed attempt to establish boundaries, protect health, reduce coercive striving, or redirect effort toward personally valued goals. Passive TP, by contrast, reflects despair-driven withdrawal that may emerge when effort and voice are perceived as ineffective and the future as difficult to influence. These functions may coexist or change over time. Importantly, the same observable behavior, doing less, may reflect either retained agency or diminished agency, and the same public statement may or may not correspond to actual withdrawal. As TP function is motive-based, it should not be inferred from discourse, self-identification, or behavior alone. A framework that fails to distinguish these underlying functions may risk conflating recovery, resistance, resignation, and psychological distress as manifestations of a single phenomenon.

The emerging evidence base has begun to move TP research beyond cultural commentary and moral interpretation. The Lying Flat Tendency Scale provides a general measure of TP tendency among Chinese youth, while the Academic Tangping Scale differentiates affective, behavioral, and cognitive manifestations within university contexts (Lu et al., 2023, 2026). Other studies have linked TP to perceived stress, anxiety, relative deprivation, basic need frustration, meaning in life, and mental health outcomes (Lu et al., 2024; Ren et al., 2026; Yang et al., 2025; Zheng et al., 2023). Although this evidence is derived largely from cross-sectional, context-specific, and self-reported research, it has established TP as a measurable and psychologically meaningful phenomenon rather than simply a sign of laziness or irresponsibility. However, existing instruments primarily capture TP tendency or manifestation; they do not yet establish whether a statement or behavior serves an active or passive function, or whether TP discourse corresponds to TP practice. This limitation raises a prior conceptual question: what, precisely, is being observed when researchers identify TP?

Once the manifestation-function distinction is made, a second unresolved question follows: what may happen when TP expression is suppressed? This article uses the term suppression of TP expression in a narrower sense than ordinary criticism, disagreement, or discouragement. It refers to institutional, media, or platform-level actions that reduce the visibility, legitimacy, searchability, or circulation of TP discourse through deletion, denunciation, algorithmic control, or stigmatizing moral narratives. Discouragement becomes suppression when it moves beyond persuasion or disagreement and involves patterned, actor-linked constraints on access, visibility, searchability, legitimacy, or circulation. This definition draws on research on Chinese internet censorship, platform visibility moderation, and public delegitimation of TP in state-linked discourse (Bamman et al., 2012; King et al., 2013; Roberts, 2018; Zeng and Kaye, 2022). At the same time, the definition leaves open the possibility that regulation varies across platforms, time periods, regions, issue salience, and degrees of perceived collective-action risk rather than treating suppression as a single, uniform national mechanism.

Existing scholarship therefore leaves two linked explanatory gaps. First, TP research often treats discourse, self-identification, and practice as if they were interchangeable, even though the motivational function of an expression may not correspond to the function of an individual's behavior. Second, censorship research typically examines whether restrictions suppress, redirect, or amplify expression, but rarely considers the prior function of the expression being constrained. Importantly, TP research has focused primarily on why young people withdraw, while paying relatively less attention to how active and passive TP expression may unfold once it is restricted. A general claim that suppression converts all visible TP into hidden disengagement would therefore be overly broad: it would overlook both the possible divergence between TP discourse and TP practice and the distinction between individuals who continue to communicate a boundary and those who already regard both speaking and striving as futile.

**Figure 1:**
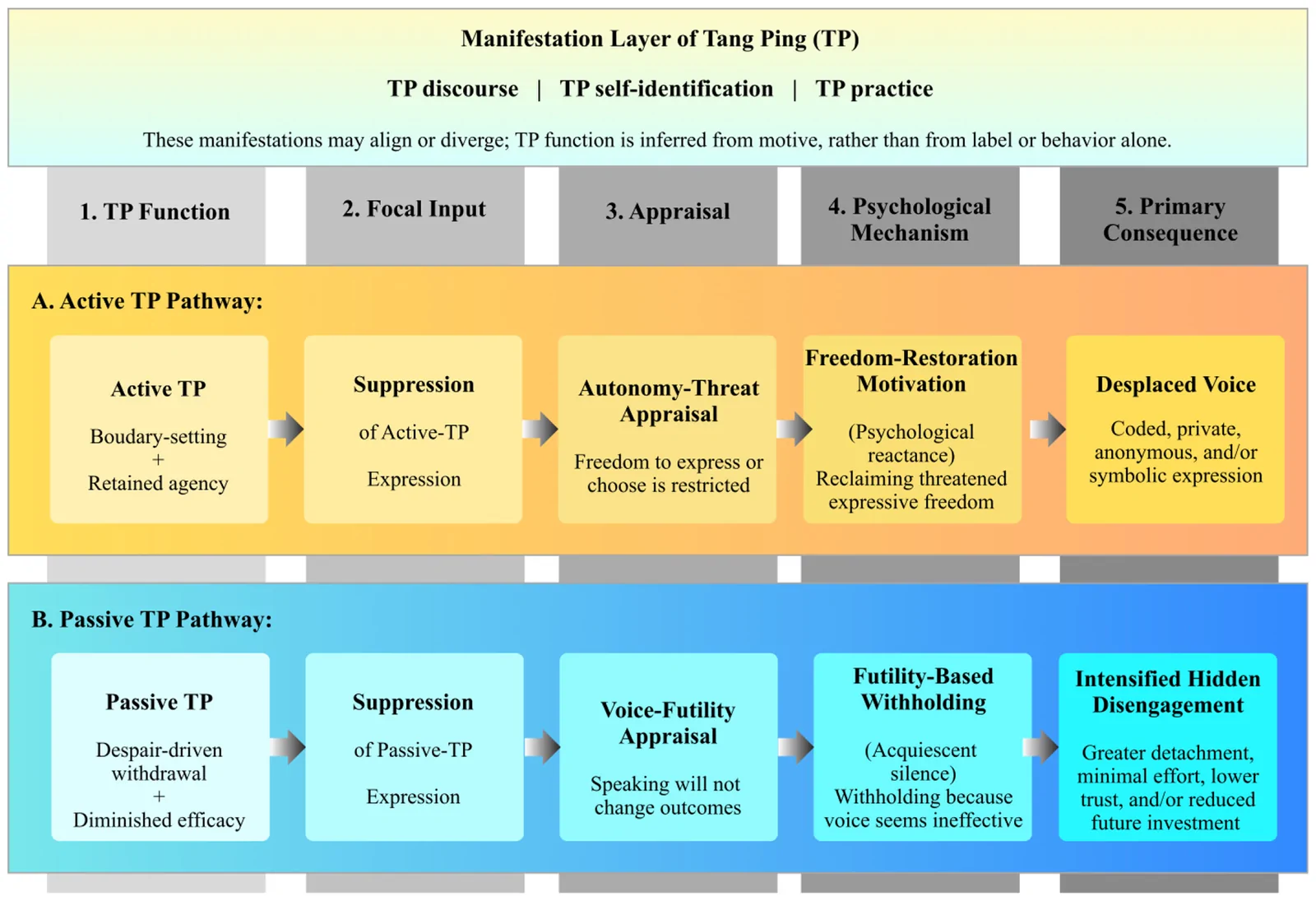
The Tang Ping Differentiated Pathways Framework (TP-DPF): manifestation-function distinction and function-contingent pathways following suppression of TP Expression. TP discourse, TP self-identification, and TP practice may align or diverge and should be measured separately. TP expression is used as the umbrella term for TP discourse and communicative self-identification, whereas TP practice refers specifically to enacted behavior. Pathway assignment depends on the function of the suppressed expression, not on label use or behavior alone. Solid arrows represent propositions rather than established causal effects.

This article aims to address these gaps through the *Tang Ping Differentiated Pathways Framework* (TP-DPF). The framework follows a two-step explanatory logic: it first separates TP manifestation from TP function and then proposes function-contingent divergence after the suppression of TP expression. Figure 1 places the manifestation layer above two comparable five-stage sequences—TP function, suppression, appraisal, psychological mechanism, and primary consequence—without assuming that the function of an expression is identical to the function of the individual's actual practice.

Having separated what is observed from the motive it may serve, the framework next traces how suppression may acquire different psychological meanings. Within this architecture, active TP expression follows a proposed voice pathway involving autonomy-threat appraisal, freedom-restoration motivation, and displaced voice, whereas passive TP expression follows a proposed withdrawal pathway involving voice-futility appraisal, futility-based withholding, and intensified hidden disengagement. The TP-DPF therefore captures a theoretically important asymmetry: suppression may redirect voice without immediately eliminating agency, or it may deepen withdrawal when agency is already weakened. Active and passive TP are treated as correlated motive-based dimensions rather than fixed types.

The framework advances existing literature in four specific respects. First, it introduces a manifestation-function distinction by separating TP discourse, TP self-identification, and TP practice from the active or passive motive that any of these manifestations may serve. Second, it extends censorship research by treating the function of restricted expression as a theoretically consequential pathway selector rather than assuming that suppression elicits a homogeneous response. Third, it extends research on youth disengagement by specifying how active and passive forms of TP expression may develop once their visibility or legitimacy is restricted. Fourth, it distinguishes observable public quiet from underlying motivational consequences: a similar decline in explicit TP discourse may reflect displaced voice in one pathway but intensified hidden disengagement in the other, while actual practice may remain unchanged, become more withdrawn, or never have matched the discourse in the first place. The function of an expression is not assumed to be identical to the function of the individual's actual practice. The remainder of the article develops this conceptual synthesis, defines the framework's core terms, situates the pathways within class-differentiated Chinese contexts, identifies cross-cultural and multilevel boundary conditions, and outlines a roadmap for empirical validation.

## 2 Conceptual synthesis, theory architecture, and conceptual boundaries

This article is a proposition-generating conceptual contribution rather than an empirical test of suppression or youth motivation. The TP-DPF was developed through a problem-driven synthesis organized around a central question: why might the suppression of the same TP label be associated with different psychological consequences, and why might those consequences fail to correspond directly to observable practice? Relevant literature was identified iteratively across six domains: empirical and conceptual research on TP; the discourse-practice relationship and social stratification; motivation and psychological needs; psychological reactance; employee voice and silence; and censorship and visibility governance. Comparative research on East Asian and Western forms of youth withdrawal was used to identify potential boundary conditions rather than to imply equivalence across contexts. Sources were retained when they clarified a manifestation of TP, the function of TP, the appraisal of suppression, a pathway-specific mechanism, a measurable consequence, or the institutional and class context within which a pathway may operate.

The manifestation-function distinction is a conceptual and measurement premise rather than an additional causal theory. Once manifestation and function have been assessed separately, three theories constitute the TP-DPF's core explanatory architecture. Self-determination theory (SDT) differentiates the initial functions of TP by distinguishing self-endorsed regulation from amotivation and by identifying autonomy and competence as psychologically consequential needs (Deci et al., 2017; Ryan and Deci, 2000). Psychological reactance theory accounts for the active pathway: when an individual with retained agency interprets suppression as a restriction of expressive freedom, motivation to restore that freedom may arise (Brehm and Brehm, 1981). Voice-and-silence theory explains the passive pathway: when an individual with low perceived efficacy interprets suppression as confirmation that speaking is unlikely to make a difference, acquiescent silence may become more likely (Knoll and van Dick, 2013; Morrison, 2023).

These three theories are integrated sequentially rather than additively, after manifestation and function have been established independently. SDT specifies the motivational condition that precedes suppression. The appraisal stage then translates suppression into a function-specific meaning, after which freedom-restoration motivation or futility-based withholding may serve as the proximal mechanism leading to a pathway-specific consequence. Reactance and silence are therefore neither alternative labels for the same response nor successive stages through which every individual must pass. Instead, they represent distinct mechanisms whose relative likelihood depends on the function of the suppressed expression and may be further shaped by perceived legitimacy, voice efficacy, institutional trust, psychological safety, access to alternative channels, and class-linked resources. A public TP statement may serve active boundary-setting even when the speaker remains unable to change actual working or family practices; conversely, an observable reduction in effort may serve a passive function without being labeled TP. To establish conceptual precision before elaborating the pathways, Table 1 summarizes the manifestations, definitions, roles, and boundaries of the core TP-DPF variables.

**Table 1:** Definitions and conceptual boundaries of the TP-DPF variables.

Role	Variable	Definition and conceptual boundary	Illustrative indicators	Core basis
Manifestation layer	TP discourse, TP self-identification, and TP practice	Three analytically distinct ways in which TP may appear: communicated claims or symbols; explicit adoption of the TP label; and observable changes in work, education, consumption, or family-related behavior. They may align or diverge and do not, by themselves, identify active or passive function.	Public or private posts; label adoption and identity centrality; work hours, participation, consumption, job or study decisions, and time use.	TP scholarship; Tong and Liu (2024).
TP function A	Active TP (boundary-setting)	A self-endorsed motive for expressing or enacting a reduction in externally imposed striving in order to protect autonomy, health, or personally valued priorities while preserving agency. Distinct from amotivation, label use, or low effort by itself.	Perceived choice; value congruence; deliberate workload limits; pursuit of alternative goals; retained response efficacy.	SDT: autonomous motivation.
TP function B	Passive TP (despair-driven withdrawal)	A motive for expressing or enacting withdrawal from socially expected striving because effort and voice are perceived as ineffective and the future as difficult to influence. Distinct from restorative rest, chosen minimalism, or behavior alone.	Low control beliefs; resignation; future pessimism; low response efficacy; withdrawal because effort seems pointless.	SDT: amotivation and competence frustration.
Focal input	Suppression of TP expression	Actor-linked restriction of the visibility, legitimacy, searchability, or circulation of function-specific TP expression. It targets discourse or self-identification directly and should be distinguished from disagreement, transparent content-neutral moderation, or the independent regulation of behavior.	Content removal; de-ranking; search non-return; account restriction; official or organizational delegitimation; anticipated penalties.	Censorship and visibility-governance scholarship.
Active appraisal	Autonomy-threat appraisal	The perception that suppression restricts the freedom to express a boundary, communicate a choice, or act consistently with self-endorsed values. It precedes reactance and is not the motivational response itself.	Perceived loss of expressive freedom; feeling controlled or voiceless; perceived illegitimacy of the restriction.	Reactance theory and SDT.
Passive appraisal	Voice-futility appraisal	The belief that speaking about strain will not influence decisions, improve conditions, or elicit a meaningful response. It concerns expected ineffectiveness rather than expected punishment.	Low expected impact of voice; low perceived responsiveness; “speaking will not change anything.”	Voice-and-silence theory.
Active mechanism	Freedom-restoration motivation (psychological reactance)	A temporary motivational response directed at reclaiming freedom perceived to have been threatened or removed. Distinct from general anger, trait oppositionality, or the preceding appraisal.	Desire to restore expressive freedom; attraction to the restricted position; intention to re-express, repost, or circumvent the restriction.	Psychological reactance theory.
Passive mechanism	Futility-based withholding (acquiescent silence)	Intentional withholding of relevant concerns because speaking is judged ineffective or pointless. Distinct from fear-based silence and from mere absence of speech.	Withholding despite holding concerns; low voice intention; deliberate non-expression because voice seems ineffective.	Voice-and-silence theory.
Active consequence	Displaced voice	Continued communication of an underlying claim through coded, private, anonymous, symbolic, indirect, or alternative channels while communicative intent remains present.	Coded language; private-group discussion; anonymous posting; memes; symbolic refusal; movement to alternative channels.	Reactance-based freedom restoration and indirect voice.
Passive consequence	Intensified hidden disengagement	A greater privately experienced reduction in psychological or behavioral investment that is not fully reflected in public expression. Distinct from burnout and depression.	Emotional detachment; minimal compliance; reduced discretionary effort; lower trust; reduced future investment.	SDT and disengagement research.

## 3 Defining suppression and avoiding circularity

Suppression of TP expression is the focal input variable in the TP-DPF, but it should be defined and measured independently of both TP practice and the proposed psychological consequences. In this framework, suppression refers to actor-linked constraints on the visibility, legitimacy, searchability, or circulation of TP expression. It does not refer directly to an individual's capacity to reduce work, study, consumption, or family obligations, although the wider climate created by suppression may influence those practices indirectly. Strong evidence may combine content-removal records, keyword and search-result audits, changes in recommendation patterns, account restrictions, official or organizational statements, and confidential user reports. A decline in the number of TP-related posts is not, by itself, sufficient evidence of suppression because public discussion may also diminish because of discourse fatigue, changing terminology, genuine improvements in underlying conditions, a shift from discourse to practice, or unrelated platform dynamics (Bamman et al., 2012; Roberts, 2018; Zeng and Kaye, 2022).

The TP-DPF therefore requires both manifestation-specific and function-specific assessment of exposure to suppression. Researchers should first establish whether the focal material is TP discourse, explicit self-identification, or a report of practice, and should then assess the motive conveyed by the expression rather than infer it from a keyword. Suppression of active-TP expression concerns messages that communicate boundary-setting, self-protection, or alternative values, whereas suppression of passive-TP expression concerns messages that communicate futility, hopelessness, or withdrawal. The speaker's actual practice should be measured separately because an active expression may coexist with continued intensive work, and a passive practice may occur without explicit TP discourse. Message coding, participant self-reports, experimental vignettes, motive-based TP measures, and behavioral indicators can be combined to determine whether the restricted expression was primarily active, passive, or mixed before the proposed pathways are examined.

The proposed consequences must likewise be measured independently of suppression, manifestation, and one another. Displaced voice cannot be inferred solely from the disappearance of public posts; it requires evidence that communicative intent persists through coded, private, anonymous, symbolic, or alternative channels. It is principally a change in the channel or form of TP discourse and does not, by itself, indicate that TP practice has intensified. Intensified hidden disengagement, by contrast, requires confidential assessment of emotional detachment, minimal compliance, reduced discretionary effort, lower institutional trust, and diminished willingness to invest in the future, whether or not the person uses the TP label. Maintaining these separations reduces circular reasoning, permits discourse-practice divergence, and renders the TP-DPF empirically falsifiable.

These distinctions also clarify how suppression may originate and operate at multiple levels. Governmental and societal actors can shape legal, political, and moral narratives; digital platforms influence visibility, searchability, and circulation; organizations and universities establish local sanctions, workload expectations, and channels for voice; and individuals interpret these conditions while facing unequal capacities to alter their actual practices. The perceived legitimacy, transparency, and proportionality of suppression, together with psychological safety, credible alternative channels, and access to economic, cultural, and social resources, can be treated as boundary conditions rather than additional mediators in the core framework. A transparent and proportionate restriction targeting demonstrably harmful content may therefore carry different implications from broad, opaque, or moralizing suppression directed at a diagnostic vocabulary through which young people communicate strain.

## 4 Active TP pathway: from autonomy threat to displaced voice

Active TP is characterized by retained agency in the motive underlying an expression or practice. Individuals may intentionally limit excessive striving, articulate an alternative conception of a worthwhile life, or publicly express a desire to establish such boundaries even when economic or relational obligations prevent them from doing so in practice. The suppression of active-TP expression may therefore elicit an autonomy-threat appraisal, whereby individuals perceive that a self-endorsed boundary, or their freedom to communicate that boundary, has been restricted (Liu et al., 2026a). This appraisal is conceptually distinct from the mechanism that follows; it captures the meaning attributed to suppression rather than the individual's subsequent response or actual level of behavioral withdrawal.

Psychological reactance theory proposes that perceived restrictions on freedom may activate motivation to restore that freedom (Brehm and Brehm, 1981). Within the TP-DPF, this mechanism is termed freedom-restoration motivation, defined as a temporary motivational state directed toward reclaiming threatened expressive freedom. Classic research on censorship suggests that restricting a message may, under some conditions, increase its attractiveness, although such responses depend on the source, legitimacy, and perceived meaning of the restriction (Worchel et al., 1975). State reactance can therefore be measured directly rather than inferred solely from anger, resistance, or non-compliance (Sittenthaler et al., 2015).

The primary consequence proposed for the active pathway is displaced voice. Although communicative intent and agency remain present, expression may shift to coded language, private groups, anonymous accounts, humor, symbolic refusal, or alternative platforms. Displaced voice is therefore a discursive consequence and should not be equated with hidden disengagement or with a completed change in practice. An individual may continue intensive work while privately criticizing it, may gradually enact stronger boundaries, or may retain the same practice because withdrawal is materially unavailable. This distinction is theoretically important because reduced public expression may otherwise be interpreted as successful silencing, whereas the active pathway suggests that voice may have been relocated without establishing either motivational recovery or behavioral withdrawal.

## 5 Passive TP pathway: from voice futility to intensified hidden disengagement

Passive TP is characterized by despair-driven withdrawal and low perceived efficacy, but this function may appear in TP discourse, TP practice, both, or neither explicit self-identification. Individuals may reduce their effort because striving appears ineffective, opportunities seem constrained, or the future is perceived as difficult to influence; some may enact such withdrawal without ever describing themselves as lying flat. When passive-TP expression is suppressed, individuals may form a voice-futility appraisal, concluding that speaking about their strain is unlikely to influence decisions, improve conditions, or generate a meaningful response. Voice futility is conceptually distinct from fear of sanction; individuals may withhold concerns because speaking appears pointless even when punishment is not anticipated. For individuals who practice withdrawal without public TP expression, the framework predicts no direct suppression effect unless they perceive the broader expressive climate or encounter suppression through related communication.

Research on employee voice and silence identifies acquiescent silence as the intentional withholding of relevant concerns, driven by resignation and low expected efficacy (Knoll and van Dick, 2013; Morrison, 2023). Within the TP-DPF, this mechanism is described in plain language as futility-based withholding, that is, the deliberate decision not to express concerns because voice is perceived as ineffective. This mechanism cannot be equated with the mere absence of speech, a stable preference for privacy, or silence motivated primarily by fear. Instead, it may reflect an intentional response to low anticipated institutional responsiveness.

The primary consequence proposed for the passive pathway is intensified hidden disengagement. Suppression is not assumed to create passive TP in the absence of prior resignation, nor is explicit TP self-identification required. Rather, suppression may deepen a privately experienced but publicly underexpressed reduction in investment among individuals for whom the relevant expression already reflects low efficacy and diminished agency. Potential indicators may include greater emotional detachment, minimal compliance, reduced discretionary effort, lower institutional trust, and weaker investment in educational, occupational, familial, or civic domains (Morrison, 2023). Because hidden disengagement concerns motivation and practice rather than label use, it should be assessed independently of public TP discourse and separately from depression and burnout. Although these constructs may coexist and overlap empirically, burnout is generally conceptualized as an occupationally situated syndrome, whereas depression extends across life domains; both can therefore be examined as alternative explanations, antecedents, or correlated outcomes rather than treated as synonymous with hidden disengagement (Maslach and Leiter, 2016; Ren et al., 2026).

## 6 What is new: manifestation-function distinction and function-contingent divergence

The TP-DPF's originality lies in a classification-and-contingency logic. Firstly, the manifestation-function distinction prevents TP discourse, TP self-identification, and TP practice from being treated as interchangeable evidence of active or passive TP. Secondly, the function-contingent divergence proposes that the motive conveyed by the suppressed expression shapes its appraisal, proximal mechanism, and primary consequence. Self-determination theory, psychological reactance theory, and voice-and-silence theory already account for motivational orientation, threatened freedom, and the withholding of voice. The TP-DPF extends these perspectives by specifying how they are connected: manifestation is observed, function is assessed independently, and function then selects the more plausible post-suppression pathway. The same suppressive intervention may therefore preserve agency while redirecting voice in one case, yet deepen withdrawal in another; moreover, the expressed pathway need not correspond directly to the individual's current practice. Neither this classification-and-contingency logic nor its discourse-practice implications are fully specified by any component theory in isolation.

This classification-and-contingency logic also changes how public quiet can be interpreted. A decline in explicit TP discourse is an observable change in visibility, but it does not resolve either of two questions: whether discourse corresponded to practice before suppression, and whether the suppressed expression served an active or passive function. Reduced public expression may coexist with continued voice through less visible channels, unchanged intensive practice, newly enacted boundaries, or intensified disengagement that is no longer openly articulated. These latent states carry different implications at organizational and societal levels. Displaced voice indicates unresolved communicative intent and migration to alternative channels, whereas intensified hidden disengagement signals declining investment, trust, and feedback. Treating all these possibilities simply as “silence” would obscure the framework's central theoretical contribution.

## 7 Chinese contextual embedding of active and passive Tang Ping

The TP-DPF is not culturally neutral. TP emerged within a distinctive moral economy of striving in which educational attainment, occupational success, homeownership, marriage, filial contribution, and national development may operate as mutually reinforcing expectations. These expectations are embedded in hypercompetitive educational and occupational environments and in a broader discourse of responsibility that links hard work, family formation, consumption, and personal contribution to collective prosperity (Zhang and Li, 2023). Structural conditions, including intensive work cultures, credential competition, hukou-related inequalities in mobility and opportunity, insecure school-to-work transitions, and housing-related pressures surrounding marriage, may further shape whether sustained effort is perceived as meaningful, attainable, and reciprocated (Chen et al., 2026; Gao et al., 2023; Wang et al., 2023; Zheng and Qiu, 2023). Although these pressures do not affect all Chinese youth uniformly, they make the meaning of “doing less” difficult to separate from family obligation, social recognition, class position, and culturally structured expectations surrounding the transition to adulthood (Gui and Koropeckyj-Cox, 2016).

Class position is especially important because it may shape the relationship between TP discourse and TP practice. Tong and Liu (2024) describe class-differentiated modes in which relatively resource-rich youth may express TP and plan a future exit while remaining embedded in competitive employment; some urban youth with family support may enact withdrawal without publicly adopting the label; and highly precarious day laborers may practice intermittent work and minimal consumption under conditions of severe constraint. Their typology should not be equated directly with the TP-DPF's active and passive functions, which are psychological rather than class categories. It nevertheless demonstrates why economic, cultural, and social resources may condition whether a stated preference can be enacted, whether practice is voluntarily sustained, and whether discourse is available or useful to the individual. The TP-DPF therefore treats class-linked resources as moderators of discourse-practice alignment and pathway strength, not as proxies for TP function.

This discourse-practice and class distinction also clarifies why the active pathway cannot be interpreted as a simple translation of Western individualism into the Chinese context. Self-determination theory distinguishes autonomy from independence. Individuals may autonomously endorse familial and collective obligations, whereas autonomy is frustrated when such obligations are experienced as coercive, humiliating, or impossible to fulfill (Chirkov et al., 2003; Kagitçibaşi, 2005). Active TP may therefore represent an effort to renegotiate relational obligations, for example, by protecting one's health, resisting excessive self-sacrifice, or redefining socially valued forms of success, rather than a rejection of family or society. When such expressions are suppressed, individuals may perceive a threat to their freedom to negotiate a socially acceptable and personally sustainable way of life. Psychological reactance may therefore remain relevant even when overt defiance is unlikely.

The same distinction also sharpens the passive pathway. Despair-driven withdrawal may reflect not only low personal control but also shame associated with failing to become the socially valued student, employee, homeowner, spouse, or family contributor prescribed by prevailing cultural narratives. When TP expression is publicly delegitimized, voice-futility appraisals may be reinforced by the expectation that institutions will interpret distress as a sign of moral weakness rather than as meaningful information about blocked opportunities. Futility-based withholding may then preserve outward conformity while private investment, institutional trust, and future orientation gradually deteriorate.

China's state-platform governance ecology further shapes how suppression is experienced and negotiated. Restrictions on visibility may arise through government communication, commercial platform moderation, organizational or educational rules, and anticipated social sanctions. Active responses may therefore take the form of homophones, memes, irony, private-group discussion, anonymous posting, or lifestyle performance rather than formal protest (King et al., 2013; Zhang and Li, 2023; Zeng and Kaye, 2022). At the same time, these processes are unlikely to operate uniformly across platforms, political periods, regions, hukou positions, occupations, genders, life stages, or levels of economic, cultural, and social capital. The framework therefore treats such differences as potential empirical moderators and avoids representing “Chinese youth” as a culturally homogeneous population.

## 8 Comparative pathways and cross-cultural applicability

International comparison is analytically important because it helps distinguish elements of the framework that may generalize across settings from those that depend on particular cultural and institutional conditions. The purpose is not to assume that all labels associated with youth withdrawal describe equivalent phenomena. Cross-cultural research cautions that similar observable behaviors may serve different psychological functions and carry different meanings across contexts (Liu, 2026; Liu et al., 2026b; Putnick and Bornstein, 2016). Instead, the TP-DPF proposes that cross-cultural comparison should first distinguish discourse, self-identification, and practice, and should then interpret suppression in relation to the function of the focal expression and the availability of credible opportunities for voice, exit, and institutional responsiveness (Morrison, 2023). Comparative cases can therefore help clarify whether similar outward behaviors represent boundary-setting, resignation, actual exit, or the postponement of major life-course transitions.

Western “quiet quitting” is generally conceptualized as a work-specific practice in which employees intentionally limit their contributions to contracted responsibilities rather than undertaking additional or extra-role work (Formica and Sfodera, 2022; Harris, 2025). It may therefore resemble active TP when employees establish occupational boundaries while retaining a sense of agency and continued attachment to their work. When employers stigmatize or penalize such boundary-setting, the active pathway suggests that perceived restrictions on freedom may be associated with psychological reactance and displaced forms of voice (Brehm and Brehm, 1981). By contrast, when repeated attempts to raise concerns are perceived as ineffective, voice-futility appraisals and acquiescent silence may become more salient (Knoll and van Dick, 2013; Morrison, 2023). The Great Resignation differs in an important respect because it involves observable exit rather than continued but less visible participation (Formica and Sfodera, 2022). It therefore illustrates how access to a credible exit option may alter or interrupt the pathways proposed by the TP-DPF.

South Korea's N-po generation and Japan's low-desire or *satori* discourses are more closely aligned with TP in their associations with employment insecurity, housing constraints, marriage, and the postponement or abandonment of conventional adulthood milestones (Chang, 2010; Matsuda et al., 2024). Nevertheless, these phenomena are embedded in different welfare institutions, labor-market structures, family regimes, and systems of public-discourse regulation. In South Korea, compressed modernity may intensify intergenerational obligations and life-course pressures by concentrating rapid socioeconomic change within family-centered systems of responsibility (Chang, 2010). Japanese low-desire narratives, by contrast, may combine deliberate non-consumption with adaptation to prolonged economic stagnation and increasingly unstable school-to-work transitions (Matsuda et al., 2024). Moreover, the regulation and perceived risks of public expression may be especially consequential in China's state-platform governance environment (King et al., 2013; Roberts, 2018). The TP-DPF therefore suggests that apparently similar labels may encompass both active and passive functions across societies, while the forms of suppression and perceived costs of public voice may differ substantially.

European youth disengagement is likewise heterogeneous across national labor-market and welfare regimes. Precarious employment, education–occupation mismatch, migration, and intergenerational disadvantage shape youth transitions differently across European societies, while labor-market policies, welfare protections, and institutionalized forms of collective representation may provide more formal opportunities for voice or exit in some settings than in others (O'Reilly et al., 2015). Cross-cultural applications of the TP-DPF can therefore assess functional equivalence, measurement invariance, and context-specific moderators before comparing latent constructs or pathway strengths across countries (Putnick and Bornstein, 2016). TP, quiet quitting, the N-po generation, “satori,” the Great Resignation, and European youth precarity should not be treated as interchangeable categories. The framework's international relevance lies in its function-contingent logic, namely, that apparently similar withdrawal behaviors may be associated with different motivational functions and institutional responses, rather than minimizing meaningful cultural and structural differences.

## 9 Multilevel embedding

Although the proposed sequences are psychological, the TP-DPF is embedded within a multilevel context. At the individual level, TP manifestation, TP function, the appraisal of suppression, the intervening psychological mechanism, and the primary consequence are experienced and reported. At the family and class-resource level, savings, housing, education, social networks, and access to support may shape whether TP discourse can be translated into practice and whether withdrawal remains voluntary or becomes constrained. At the organizational and university levels, workload demands, procedural fairness, local leadership, and psychological safety shape the extent to which concerns can be expressed credibly. At the digital-platform level, searchability, recommendation systems, de-ranking practices, account sanctions, and appeal procedures determine the visibility and circulation of TP expression. At the governmental and societal levels, employment conditions, housing pressures, educational systems, developmental narratives, and the perceived legitimacy of dissent shape both the structural strains associated with TP and the meaning attributed to its suppression.

These cross-level influences can be specified as moderators rather than incorporated as additional mediators in the core pathways. Economic, cultural, and social resources may moderate the correspondence between TP discourse and TP practice, while perceived legitimacy and transparency may weaken autonomy-threat appraisals and credible voice channels and psychological safety may reduce voice-futility appraisals and futility-based withholding (Edmondson, 1999). Institutional trust may shape both pathways: low trust may increase the likelihood that suppression is interpreted as illegitimate in the active pathway while simultaneously reducing expectations of institutional responsiveness in the passive pathway. Material insecurity may further restrict the feasibility of active TP practice, increase the costs of displaced voice, and intensify the consequences of hidden disengagement. Together, these moderators delineate the conditions under which discourse and practice are more likely to align, and the proposed pathways are more likely to be strengthened, weakened, or absent.

## 10 Operational propositions and summary table

The propositions follow the classification-and-contingency logic of Figure 1 and use independently measurable variables. They first distinguish TP manifestation from TP function and then test the function-contingent pathways associated with the suppression of TP expression. They are theoretical expectations rather than empirical conclusions. Table 2 consolidates the independent variable, mediator, outcome, theoretical basis, and suggested test, enabling evaluation of the framework without treating discourse, self-identification, and practice as interchangeable.

**Table 2:** Summary of TP-DPF propositions.

Proposition	Independent variable	Mediator	Outcome	Theory	Suggested test
P1: Manifestation-function differentiation	TP discourse, TP self-identification, TP practice, economic/cultural/social resources, autonomous motivation, competence frustration, amotivation, and response efficacy.	No serial mediator; TP manifestation and TP function are modeled as distinct constructs.	Discourse, self-identification, and practice will be empirically distinguishable and only imperfectly aligned; resource access will strengthen the translation of stated preferences into practice. Active TP will be associated more strongly with retained choice and autonomous motivation, whereas passive TP will be associated more strongly with competence frustration, amotivation, and low response efficacy.	TP scholarship and SDT.	Multitrait-multimethod CFA/ESEM, latent profile analysis, and moderation of discourse-practice alignment.
P2: Active pathway	Perceived or manipulated suppression of independently classified active-TP expression.	Autonomy-threat appraisal → freedom-restoration motivation.	Displaced voice through coded, private, anonymous, symbolic, or alternative channels.	Psychological reactance theory, with SDT specifying retained agency.	Preregistered message-suppression experiment and longitudinal moderated serial mediation.
P3: Passive pathway	Perceived or manipulated suppression of independently classified passive-TP expression.	Voice-futility appraisal → futility-based withholding.	Intensified hidden disengagement: detachment, minimal effort, lower trust, and reduced future investment.	Voice-and-silence theory, with SDT specifying low efficacy and amotivation.	Three-wave longitudinal serial mediation; experimental manipulation of responsiveness.
P4: Divergent indirect effects	Suppression × TP function, with TP manifestation and actual practice modeled separately.	Active and passive serial mechanisms modeled simultaneously.	The indirect association with displaced voice will be stronger when the suppressed expression serves active TP; the indirect association with hidden disengagement will be stronger when it serves passive TP, irrespective of whether the speaker's current practice matches the expression.	Integrated TP-DPF.	Multigroup or latent-interaction SEM; planned contrast of conditional indirect effects.
P5: Contextual moderation	Perceived legitimacy, alternative voice channels, psychological safety, institutional trust, suppression source, class-linked resources, and discourse-practice alignment.	Moderation of appraisal and mechanism paths.	Weaker pathway associations when suppression is transparent and legitimate, safe and credible voice channels remain available, and class-linked resources permit meaningful boundary-setting or alternative action.	Reactance and voice-and-silence theories.	Multilevel SEM across organizations, universities, regions, or platforms.
P6: Downstream implications	Displaced voice and intensified hidden disengagement measured separately.	No required mediator in the core model.	Displaced voice will predict channel migration and continued communicative intent; hidden disengagement will predict lower wellbeing, trust, discretionary contribution, and future investment.	TP-DPF extension to organizational and societal outcomes.	Multiwave outcome models using behavioral and administrative indicators where ethical.

## 11 Measurement, sampling, and analytical roadmap

Table 3 consolidates the proposed empirical validation roadmap by linking the manifestation-function distinction and each core TP-DPF pathway to appropriate measures, sampling requirements, research designs, and analytical approaches. The ordering is deliberate: researchers should establish what form of TP is observed and what function it serves before testing the consequences of suppressing its expression.

**Table 3:** Empirical validation roadmap.

Objective	Sampling/design	Measures	Primary analysis
TP-manifestation and function validation	30–50 qualitative interviews; EFA 400–600; independent CFA 600–1,000.	Lying Flat Tendency Scale; Academic Tangping Scale; content and self-identification measures; behavioral practice indicators; new TP Function Module; class-resource measures.	Multitrait-multimethod ESEM/CFA, latent profile analysis, discourse-practice alignment models, and invariance testing.
Proximal experimental responses	Preregistered 2 × 2 or 2 × 2 × 2 vignette/platform experiment; final N from simulation, often several hundred.	Validated active/passive message content; suppression manipulation; baseline TP practice; appraisal scales; SSR; voice-efficacy and acquiescent-silence items; behavioral channel choices.	Factorial regression or SEM; moderated serial mediation; planned contrasts.
Temporal pathways	Three or four waves; baseline approximately 1,200–1,500 across multiple settings.	Repeated measures of TP discourse, self-identification, practice, function, suppression, pathway variables, and independently measured outcomes.	RI-CLPM or latent change modeling; longitudinal SEM; missing-data sensitivity analyses.
Cross-level context	Individuals nested in organizations, universities, regions, or platforms.	Psychological safety, legitimacy, documented moderation, employment insecurity, institutional trust, and economic, cultural, and social resources.	Multilevel SEM and cross-level moderation.
Cross-cultural scope	Matched samples in China, South Korea, Japan, North America, and European settings.	Translated and culturally adapted measures with manifestation- and function-equivalent scenarios.	Configural, metric, and scalar invariance; comparative mixed methods.

### 11.1 Measurement strategy

Measurement should proceed in two steps. First, researchers should identify the manifestation of TP: discourse can be assessed through message content, channel, audience, and frequency; self-identification through explicit label adoption and identity centrality; and practice through time use, work or study participation, consumption, job transitions, and other behavioral indicators. Second, researchers should assess the active or passive function of the focal expression or practice with motive-based items. Parallel questions about what participants say and what they do are necessary because one person may display active TP discourse while remaining behaviorally constrained, whereas another may display passive TP practice without using the TP label.

After TP manifestations have been recorded separately, the function served by each focal manifestation needs to be assessed before estimating the proposed pathways. The Lying Flat Tendency Scale can provide a general measure of TP tendency, whereas the Academic Tangping Scale can assess affective, cognitive, and behavioral manifestations in student populations (Lu et al., 2023, 2026). However, neither instrument directly determines whether similar expressions or practices are motivated by boundary-setting or despair. A dedicated TP Function Module can therefore be developed through qualitative item generation and cognitive interviewing. Items assessing the active function should capture self-endorsed boundary-setting, value congruence, recovery, retained choice, and intended alternative goals. Items assessing the passive function should capture futility, low perceived control, resignation, and a constricted view of the future. Exploratory structural equation modeling and latent profile analysis could then examine whether active, passive, mixed, and low-TP functions emerge within discourse and practice separately, and whether expressive-only, practice-only, aligned, and divergent manifestation profiles can be identified.

Basic psychological needs can be assessed using the Basic Psychological Need Satisfaction and Frustration Scale, which distinguishes the satisfaction and frustration of autonomy, competence, and relatedness (Chen et al., 2015). Suppression requires a context-specific measure covering experienced restrictions on visibility, public delegitimation, anticipated sanctions, transparency, and perceived legitimacy. Where feasible, self-reported exposure can be triangulated with search audits, platform traces, or documented moderation practices. Economic, cultural, and social resources should be measured through indicators such as income and savings, housing and family support, educational credentials, occupational position, and access to networks, because these resources may condition the translation of TP discourse into practice (Tong and Liu, 2024). In experimental research, function-specific vignettes should establish that participants interpret a TP message as reflecting active boundary-setting or passive withdrawal before a suppression manipulation is introduced; the participant's own TP practice should be measured separately rather than inferred from the assigned message.

Autonomy-threat appraisal should be assessed separately from psychological reactance, using items that measure perceived restrictions on expressive freedom and perceived external control. Freedom-restoration motivation may be assessed using state-reactance instruments, such as the *Salzburger State Reactance Scale*, supplemented by behavioral indicators including decisions to repost, recode a message, or migrate to an alternative communication channel (Sittenthaler et al., 2015). Voice-futility appraisal requires context-specific items assessing expected institutional responsiveness and the anticipated influence of speaking up. Futility-based withholding may be measured using the acquiescent-silence dimension developed by Knoll and van Dick (2013), although adaptations beyond formal workplace settings should be undertaken cautiously and evaluated for measurement invariance.

Displaced voice should preferably be assessed through behavioral indicators, including the selection of coded forms of expression, anonymous or private communication channels, diary reports, and digital traces, rather than through attitudinal measures alone. Because it is a discourse-level consequence, displaced voice should be analyzed separately from changes in work, study, consumption, or family practice. Intensified hidden disengagement can be modeled as a multidimensional outcome encompassing emotional detachment, minimal compliance, reduced discretionary effort, lower institutional trust, and diminished future investment. The UWES-9 may provide a validated indicator of engagement in occupational or educational settings, but low engagement cannot be treated as either the sole measure or a simple inverse of hidden disengagement (Schaufeli et al., 2006). Depression, anxiety, burnout, and hopelessness can be assessed as potential antecedents, alternative explanations, or correlated outcomes rather than incorporated into the definition of hidden disengagement. Where ethical and feasible, behavioral indicators should be used to examine whether TP discourse and TP practice actually converge over time.

### 11.2 Sampling strategy

A comprehensive validation program needs to extend beyond a single convenience sample of university students and beyond participants who publicly use the TP label. Within China, stratified recruitment can include university and vocational students, early-career professionals, manufacturing and service-sector employees, platform workers, unemployed graduates, and young adults who are not engaged in stable education or employment. Sampling should deliberately capture participants who express TP but cannot enact it, those who enact withdrawal without self-identifying as TP, those for whom discourse and practice align, and those who show neither manifestation. It should also capture variation across eastern, central, and western regions; urban and rural backgrounds; hukou status; gender; educational attainment; occupation; employment security; socioeconomic position; family support; and patterns of platform use. Oversampling groups with weaker institutional attachment or limited digital visibility may be particularly important because passive disengagement and practice-only TP could both reduce the likelihood of research participation.

For initial construct development, approximately 30–50 maximum-variation and cognitive interviews may provide a reasonable illustrative planning range, although adequacy should be evaluated in terms of information power rather than a mechanical saturation threshold (Malterud et al., 2016). An exploratory sample of approximately 400–600 participants, followed by an independent confirmatory sample of approximately 600–1,000 participants, could support factor, latent profile, and measurement invariance analyses for a moderately complex instrument. These figures can be treated as illustrative planning ranges rather than universal requirements. Final sample sizes can be preregistered and justified through Monte Carlo power analyses that account for factor loadings, indirect relationships, clustering, attrition, and missing data (Muthén and Muthén, 2002; Wolf et al., 2013).

A full longitudinal investigation could begin with approximately 1,200–1,500 participants recruited across multiple universities, workplaces, regions, or platform-user groups to accommodate attrition and permit multilevel estimation. At least three waves would be needed to temporally separate suppression, appraisals or mechanisms, and proposed consequences, whereas four waves would provide a stronger basis for examining reciprocal relationships and changes in TP function. A preregistered experiment could employ a 2 × 2 design crossing active vs. passive TP message content with suppression vs. visible-expression conditions. An additional manipulation of perceived legitimacy or institutional responsiveness could be included where statistical power permits. Cross-cultural studies can recruit functionally comparable samples and establish measurement invariance before comparing latent means or structural-path coefficients.

### 11.3 Analytical strategy

The first analytical priority is to establish two related forms of discriminant validity. A multitrait-multimethod confirmatory factor model or exploratory structural equation model can test whether TP discourse, TP self-identification, and TP practice are distinguishable manifestations, while a separate motive-based model can test whether active TP and passive TP are distinguishable functions within those manifestations. Latent profile analysis may then identify expressive-only, practice-only, aligned, and divergent configurations, as well as active, passive, mixed, and low-TP functions. Measurement invariance can be evaluated across students and employees, socioeconomic groups, regions, genders, and national contexts.

Only after manifestation and function have been distinguished should the active and passive serial pathways be estimated within the same model. Moderated serial-mediation analyses could examine whether active TP function strengthens the proposed indirect association between suppression of TP expression and displaced voice, whereas passive TP function strengthens the proposed indirect association between suppression and intensified hidden disengagement. TP practice should be included as a separate time-varying variable rather than treated as evidence of pathway membership. Planned contrasts can compare the conditional indirect associations across pathways, and interactions can test whether class-linked resources moderate discourse-practice alignment or pathway strength. Cross-sectional mediation analyses need to be interpreted as exploratory because they may yield biased estimates of processes that are theoretically longitudinal (Maxwell and Cole, 2007).

For longitudinal data, random-intercept cross-lagged panel models or latent change models may help distinguish stable between-person differences from within-person change, provided that their assumptions are theoretically and empirically defensible (Hamaker et al., 2015). Multilevel SEM is appropriate when participants are nested within organizations, universities, regions, or platform environments. Relevant cross-level predictors may include documented moderation intensity, psychological-safety climate, local employment conditions, and the availability of credible voice channels. Qualitative interviews, diary studies, and digital ethnography can complement quantitative analyses by clarifying the meanings of coded expressions and assessing whether standardized measures adequately represent participants' lived experiences.

## 12 Discussion: theoretical, practical, and societal implications

### 12.1 Theoretical implications

The TP-DPF reframes the central question from “Does suppression cause disengagement?” to “What has been suppressed, what function did that expression serve, and which psychological pathway may follow?” This reframing makes the framework's originality more precise. The TP-DPF is not novel because it places self-determination theory, psychological reactance theory, and voice-and-silence theory side by side. The TP-DPF advances a classification-and-contingency logic: it first separates what is observed from the motive it may serve and then proposes that this motive conditions the psychological pathway associated with suppression. The framework thereby extends censorship scholarship by proposing heterogeneous responses to the same restriction and extends youth-disengagement research by specifying post-suppression trajectories without assuming that public discourse directly represents private motivation or observable practice.

The framework also uses its key terms consistently and assigns each a single analytical role. TP discourse, TP self-identification, and TP practice are manifestations; active TP and passive TP are motive-based functions; autonomy-threat appraisal and voice-futility appraisal are appraisals; freedom-restoration motivation and futility-based withholding are mechanisms; and displaced voice and intensified hidden disengagement are proposed consequences. Active and passive TP should not be understood as fixed personality types, nor should either pathway be regarded as inevitable. Individuals may display mixed functions, differ across discourse and practice, move between active and passive forms over time, or show little response when suppression is perceived as legitimate or when credible alternative channels remain available. These qualifications enhance the framework's falsifiability and prevent it from becoming an overly encompassing explanation of youth withdrawal.

### 12.2 Practical and policy implications

The differentiated pathways and the manifestation-function distinction imply the need for more careful diagnosis before intervention. Public endorsement of TP should not be treated as proof that a person has withdrawn in practice, and the absence of TP language should not be treated as proof of continued engagement. Active TP calls for dialogue concerning boundaries, workload, values, and alternative forms of contribution. Workplaces and universities may respond by establishing credible workload limits, participatory decision-making processes, non-retaliatory channels for voice, and visible follow-through after concerns are raised. Suppressing boundary-setting while leaving excessive demands unchanged may merely redirect voice rather than restore commitment. Managers and educators should therefore assess both communicative intent and actual behavior instead of treating all TP expression or all reductions in effort as the same problem.

Passive TP requires the restoration of efficacy as well as opportunities for expression, including for individuals who enact withdrawal without adopting the TP label. Relevant responses may include realistic pathways into employment and advancement, fairer relationships between effort and reward, accessible mental health support, mentoring, and credible evidence that speaking up can lead to a meaningful response. At the policy level, youth employment security, housing affordability, and transparent mechanisms for participation are more directly connected to perceived futility than motivational campaigns alone. Because resources affect whether a preference for reduced striving can become a sustainable practice, interventions should also avoid romanticizing “choice” where withdrawal is produced by exclusion or severe constraint. Hope is unlikely to become credible through exhortation when the institutional pathways linking sustained effort to adult security remain weak.

For digital platforms and public institutions, the framework does not imply that all forms of moderation are illegitimate. Rather, it suggests that broad or opaque suppression of TP expression may carry different costs depending on the function of the expression, while keyword-based monitoring may miss practice-only withdrawal entirely. Proportionate moderation should specify the harm being addressed, distinguish disclosures of despair from genuinely harmful incitement, explain visibility decisions where feasible, provide accessible appeal procedures, and assess private wellbeing and actual participation rather than treating reduced keyword visibility as evidence of recovery. Public dialogue is valuable not because every claim is necessarily accurate, but because it preserves information about whether young people are expressing unrealized boundaries, enacting alternative practices, or losing confidence in their capacity to influence their circumstances.

### 12.3 Alternative explanations and limitations

Suppression cannot be assumed to be the primary explanation for hidden disengagement or for divergence between TP discourse and TP practice. Depressive symptoms, burnout, perfectionism, socioeconomic disadvantage, family obligations, technological changes in labor markets, and post-pandemic adjustment may independently contribute to expression, withdrawal, or both. Class-linked resources may also explain why some individuals can enact chosen boundary-setting while others can only express it symbolically or withdraw under constraint. These factors may function as antecedents, confounders, parallel mechanisms, or vulnerability conditions. Rigorous empirical tests can therefore control for baseline mental health and disengagement, assess structural and resource conditions directly, and compare the TP-DPF with plausible alternative models.

The framework is conceptual and has not yet received direct empirical validation. Active and passive TP require motive-based measures that extend beyond existing general or domain-specific scales, and the distinction among TP discourse, TP self-identification, and TP practice also requires validation across class and institutional contexts. Research on politically sensitive expression faces challenges related to sample selection, social desirability, platform access, and participant safety; practice-only withdrawal may be especially difficult to recruit, while public expression may overrepresent groups with greater cultural or digital capital. Some constructs may overlap empirically, and the two proposed pathways may not exhaust all possible responses. Fear-based silence, genuine attitude change, unchanged behavior, and successful institutional repair are among the plausible alternatives. Cross-cultural application likewise requires caution because autonomy, voice, withdrawal, class resources, and suppression may not have equivalent meanings across institutional and cultural settings.

### 12.4 Long-term organizational and societal consequences

The two proposed consequences imply different long-term risks, but those risks should not be inferred directly from TP discourse. Displaced voice may preserve agency and retain some diagnostic information, yet the individual may remain fully engaged in intensive practice because withdrawal is unavailable; alternatively, communication may fragment across private, coded, or alternative spaces that institutions find difficult to interpret. Intensified hidden disengagement may reduce discretionary contribution, organizational learning, educational participation, and willingness to invest in future occupational, familial, or civic projects, including among individuals who never use the TP label. If sufficiently widespread, these patterns may be associated with lower innovation, productivity, institutional trust, and social cohesion. Such aggregate implications remain theoretical expectations and cannot be inferred from individual silence, TP keyword trends, or demographic patterns alone.

The multilevel implication is therefore primarily diagnostic. Governments, organizations, universities, and digital platforms may observe a quieter public environment while confronting markedly different underlying conditions: discourse may have moved elsewhere, stated preferences may remain behaviorally unrealized, practice-only withdrawal may be invisible to keyword monitoring, or private investment may be deteriorating. Administrative data may reveal employment, attendance, turnover, or service-use patterns, but they may not fully capture the meanings young people attach to effort, fairness, and the credibility of the future. The policy challenge is not simply to increase or reduce expression, but to preserve sufficient diagnostic capacity to distinguish discourse from practice and redirected voice from deepening withdrawal.

## 13 Conclusion

The theoretical contribution of the TP-DPF lies in two linked distinctions. First, the manifestation-function distinction separates TP discourse, TP self-identification, and TP practice from the active or passive motive that any of them may serve. Second, function-contingent divergence proposes that suppressing active-TP expression may be associated with an autonomy-threat appraisal, freedom-restoration motivation, and displaced voice, whereas suppressing passive-TP expression may be associated with a voice-futility appraisal, futility-based withholding, and intensified hidden disengagement. The framework's originality lies in connecting these distinctions through a parsimonious selection logic: manifestation is observed, function is assessed independently, and function then shapes the pathway that may follow suppression. Its practical contribution is to distinguish boundary-setting from resignation without assuming that discourse and practice coincide. Its policy contribution is the proposition that opportunities for voice, structural reform, mental health support, and material resources should be tailored to the function and practicability of TP rather than replaced by efforts to control its expression. Its contribution to future research is an operational roadmap for measuring the proposed constructs independently and examining their relationships through longitudinal, experimental, comparative, and multilevel designs.

The framework does not establish that suppression has already produced either pathway among Chinese youth, that public TP discourse reliably predicts TP practice, or that class position determines psychological function. Instead, it offers a cautious and falsifiable explanation of why the same observable outcome, a decline in explicit TP discourse, may conceal different psychological and behavioral realities. Its central claim is deliberately bounded: when lying flat is silenced, what follows may depend on what was expressed, whether that expression could be enacted, and whether it reflected an attempt to communicate a boundary or an emerging belief that neither striving nor speaking is likely to make a difference.

## Data Availability

The original contributions presented in the study are included in the article/supplementary material, further inquiries can be directed to the corresponding author.

## References

[B1] Bamman D. O'Connor B. Smith N. A. (2012). Censorship and deletion practices in Chinese social media. First Monday 17. doi: 10.5210/fm.v17i3.3943

[B2] Brehm S. S. Brehm J. W. (1981). Psychological Reactance: A Theory of Freedom and Control. New York, NY: Academic Press.

[B3] Chang K. S. (2010). South Korea under Compressed Modernity: Familial Political Economy in Transition. Abingdon: Routledge.

[B4] Chen B. Vansteenkiste M. Beyers W. Boone L. Deci E. L. Van der Kaap-Deeder J. . (2015). Basic psychological need satisfaction, need frustration, and need strength across four cultures. Motiv. Emot. 39, 216–236. doi: 10.1007/s11031-014-9450-1

[B5] Chen B. H. Y. Zhou Y. Yang L. (2026). The role of involution on Chinese youth pursuit of postgraduate education abroad: examine through the lens of normative social behaviour. Asia Pac. J. Educ. 46, 905–918. doi: 10.1080/02188791.2024.2408394

[B6] Chirkov V. I. Ryan R. M. Kim Y. Kaplan U. (2003). Differentiating autonomy from individualism and independence: a self-determination theory perspective on internalization of cultural orientations and well-being. J. Pers. Soc. Psychol. 84, 97–110. doi: 10.1037/0022-3514.84.1.97 12518973

[B7] Deci E. L. Olafsen A. H. Ryan R. M. (2017). Self-determination theory in work organizations: the state of a science. Annu. Rev. Organ. Psychol. Organ. Behav. 4, 19–43. doi: 10.1146/annurev-orgpsych-032516-113108

[B8] Edmondson A. (1999). Psychological safety and learning behavior in work teams. Adm. Sci. Q. 44, 350–383. doi: 10.2307/2666999

[B9] Formica S. Sfodera F. (2022). The great resignation and quiet quitting paradigm shifts: an overview of current situation and future research directions. J. Hosp. Market. Manag. 31, 899–907. doi: 10.1080/19368623.2022.2136601

[B10] Gao Y. Alessie R. Angelini V. (2023). Parental housing wealth and children's marriage prospects in China—Evidence from CHARLS. Rev. Econ. Household 21, 615–644. doi: 10.1007/s11150-022-09608-8

[B11] Gui T. Koropeckyj-Cox T. (2016). “I am the only child of my parents”: perspectives on future elder care for parents among Chinese only-children living overseas. J. Cross-Cult. Gerontol. 31, 255–275. doi: 10.1007/s10823-016-9295-z 27236539

[B12] Hamaker E. L. Kuiper R. M. Grasman R. P. P. P. (2015). A critique of the cross-lagged panel model. Psychol. Methods 20, 102–116. doi: 10.1037/a0038889 25822208

[B13] Harris L. C. (2025). Commitment and quiet quitting: a qualitative longitudinal study. Hum. Resourc. Manag. 64, 565–582. doi: 10.1002/hrm.22274

[B14] Hsu H. Y. (2022). How do Chinese people evaluate “Tang-Ping” (lying flat) and effort-making: the moderation effect of return expectation. Front. Psychol. 13:871439. doi: 10.3389/fpsyg.2022.871439 36467191 PMC9709313

[B15] Kagitçibaşi Ç. (2005). Autonomy and relatedness in cultural context: implications for self and family. J. Cross Cult. Psychol. 36, 403–422. doi: 10.1177/0022022105275959

[B16] King G. Pan J. Roberts M. E. (2013). How censorship in China allows government criticism but silences collective expression. Am. Polit. Sci. Rev. 107, 326–343. doi: 10.1017/S0003055413000014

[B17] Knoll M. van Dick R. (2013). Do I hear the whistle…? A first attempt to measure four forms of employee silence and their correlates. J. Bus. Ethics 113, 349–362. doi: 10.1007/s10551-012-1308-4

[B18] Liu H. J. (2026). Viewing ethnocentrism and ethnorelativism through a two-factor lens: a dual-spectrum model for intercultural development. Int. J. Educ. Cult. Soc. 11, 24–33. doi: 10.11648/j.ijecs.20261102.11

[B19] Liu H. J. de Jonge K. M. M. den Hartigh R. J. R. Van Yperen N. W. (2026a). Basic psychological need support, need satisfaction, and autonomous motivation in coach–athlete relationships: a systematic review and meta-analysis. Int. J. Sports Sci. Coach. 21, 1178–1191. doi: 10.1177/17479541251400621

[B20] Liu H. J. Den Hartigh R. J. R. van der Poel M. H. (2026b). Understanding the developmental model of intercultural sensitivity through a complex dynamic systems lens. Cult. Psychol. doi: 10.1177/1354067X261434563

[B21] Lu H. Hou J. Huang A. Wang J. Kong F. (2023). Development and validation of the “Lying Flat” tendency scale for the youth. Behav. Sci. 13:915. doi: 10.3390/bs13110915 37998662 PMC10669194

[B22] Lu H. Hou J. Wang J. Kong F. (2024). Antidote or poison: the relationship between “lying flat” tendency and mental health. Appl. Psychol. Health Well-Being 16, 1757–1777. doi: 10.1111/aphw.12556 38775377

[B23] Lu S. Yang Y. Li W. Jing B. Guo Y. (2026). Academic Tangping scale for college students in China: scale development, validation and application. Sci. Rep. 16:7897. doi: 10.1038/s41598-026-38759-2 41663561 PMC12954069

[B24] Malterud K. Siersma V. D. Guassora A. D. (2016). Sample size in qualitative interview studies: guided by information power. Qual. Health Res. 26, 1753–1760. doi: 10.1177/1049732315617444 26613970

[B25] Maslach C. Leiter M. P. (2016). Understanding the burnout experience: recent research and its implications for psychiatry. World Psychiatry 15, 103–111. doi: 10.1002/wps.20311 27265691 PMC4911781

[B26] Matsuda S. Sasaki T. Shin J. Bae J. (2024). Deterioration in youth employment, social contexts, and marriage decline in Japan and South Korea. Asian Popul. Stud. 20, 203–221. doi: 10.1080/17441730.2023.2211402

[B27] Maxwell S. E. Cole D. A. (2007). Bias in cross-sectional analyses of longitudinal mediation. Psychol. Methods 12, 23–44. doi: 10.1037/1082-989X.12.1.23 17402810

[B28] Morrison E. W. (2023). Employee voice and silence: taking stock a decade later. Annu. Rev. Organ. Psychol. Organ. Behav. 10, 79–107. doi: 10.1146/annurev-orgpsych-120920-054654

[B29] Muthén L. K. Muthén B. O. (2002). How to use a Monte Carlo study to decide on sample size and determine power. Struct. Equ. Model. Multidiscip. J. 9, 599–620. doi: 10.1207/S15328007SEM0904_8

[B30] O'Reilly J. Eichhorst W. Gábos A. Hadjivassiliou K. Lain D. Leschke J. . (2015). Five characteristics of youth unemployment in Europe: flexibility, education, migration, family legacies, and EU policy. SAGE Open 5, 1–19. doi: 10.1177/2158244015574962

[B31] Putnick D. L. Bornstein M. H. (2016). Measurement invariance conventions and reporting: the state of the art and future directions for psychological research. Dev. Rev. 41, 71–90. doi: 10.1016/j.dr.2016.06.004 27942093 PMC5145197

[B32] Ren X. Abdullah H. Shaffril H. A. M. Rahman H. A. Zaremohzzabieh Z. (2026). A scoping review of “Tang ping” (lying flat) and mental health status among Chinese youth. PLoS ONE 21:e0342591. doi: 10.1371/journal.pone.0342591 41805794 PMC12974857

[B33] Roberts M. E. (2018). Censored: Distraction and Diversion Inside China's Great Firewall. Princeton, NJ: Princeton University Press.

[B34] Ryan R. M. Deci E. L. (2000). Self-determination theory and the facilitation of intrinsic motivation, social development, and well-being. Am. Psychol. 55, 68–78. doi: 10.1037/0003-066X.55.1.68 11392867

[B35] Schaufeli W. B. Bakker A. B. Salanova M. (2006). The measurement of work engagement with a short questionnaire: a cross-national study. Educ. Psychol. Meas. 66, 701–716. doi: 10.1177/0013164405282471

[B36] Sittenthaler S. Traut-Mattausch E. Steindl C. Jonas E. (2015). Salzburger State Reactance Scale (SSR Scale): validation of a scale measuring state reactance. Z. Psychol. 223, 257–266. doi: 10.1027/2151-2604/a000227 27453806 PMC4957539

[B37] Sun L. (2025). Beyond resistance and resignation: a narrative inquiry into the “lying flat” movement and the reimagining of work, success, and autonomy among Chinese urban youth. Asian J. Soc. Sci. 53:100198. doi: 10.1016/j.ajss.2025.100198

[B38] Tong X. Liu J. (2024). “Lying flat”: Chinese youths' resistance to overwork and the practice of stratification. Youth Glob. 5, 231–251. doi: 10.1163/25895745-05020002

[B39] Wang Q. Cui C. Yu C. Wang Y. (2023). From domicile to university to work: the sequential migration of young educated people in the context of the “battle for talent” in China. Popul. Res. Policy Rev. 42:92. doi: 10.1007/s11113-023-09838-3

[B40] Wolf E. J. Harrington K. M. Clark S. L. Miller M. W. (2013). Sample size requirements for structural equation models: an evaluation of power, bias, and solution propriety. Educ. Psychol. Meas. 73, 913–934. doi: 10.1177/0013164413495237 25705052 PMC4334479

[B41] Worchel S. Arnold S. E. Baker M. (1975). The effects of censorship on attitude change: the influence of censor and communication characteristics. J. Appl. Soc. Psychol. 5, 227–239. doi: 10.1111/j.1559-1816.1975.tb00678.x

[B42] Yang L. Zhang Y. Zhang D. Shen L. Feng H. (2025). Association between active/passive involution and lying flat among college students: the mediation role of perceived stress and anxiety. BMC Public Health 25:1355. doi: 10.1186/s12889-025-21994-z 40452040 PMC12128238

[B43] Zeng J. Kaye D. B. V. (2022). From content moderation to visibility moderation: a case study of platform governance on TikTok. Policy Internet 14, 79–95. doi: 10.1002/poi3.287

[B44] Zhang L. Hui B. P. H. Kong F. Lu H. Chen S. X. (2025). Why people “lie flat”? An integrative framework of social-psychological pathways in China. Pers. Soc. Psychol. Rev. 29, 371–382. doi: 10.1177/10888683251358516 40728129

[B45] Zhang Z. Li K. (2023). So you choose to “lie flat?” “Sang-ness,” affective economies, and the “lying flat” movement. Q. J. Speech 109, 48–69. doi: 10.1080/00335630.2022.2143549

[B46] Zheng X. Jing C. Liu Y. Zhang Y. Y. (2023). Why are people “lying flat”? Personal relative deprivation suppresses self-improvement motivation. Br. J. Soc. Psychol. 62, 932–948. doi: 10.1111/bjso.12611 36453146

[B47] Zheng X. Qiu Z. (2023). The 996 working pattern in Chinese internet firms: how hegemonic despotism promotes long working hours for employees. China Perspect. 134, 67–78. doi: 10.4000/chinaperspectives.15869

